# The role of podocyte damage in the etiology of ischemia-reperfusion acute kidney injury and post-injury fibrosis

**DOI:** 10.1186/s12882-019-1298-x

**Published:** 2019-03-28

**Authors:** Yi Chen, Liyu Lin, Xuan Tao, Yankun Song, Jiong Cui, Jianxin Wan

**Affiliations:** 1 Department of Nephrology, The First Affiliated Hospital of Fujian Medical University, Fuzhou, 350005 China; 2 Department of Pathology, The First Affiliated Hospital of Fujian Medical University, Fuzhou, China

**Keywords:** Podocyte, Acute kidney injury (AKI), Chronic kidney disease (CKD), Synaptopodin, Nephrin, CD2AP, TRPC6

## Abstract

**Background:**

To establish a model of chronic renal fibrosis following acute kidney injury (AKI) in BALB/c mice and to observe the effect of AKI on podocyte injury and chronic fibrosis of the kidney. Additional aims included using the model to explore the role of podocyte injury in AKI and post-injury fibrosis.

**Methods:**

Fifty BALB/C mice were randomly divided into control group (Ctr), sham group (sham), AKI 20 group (renal ischemia, 20 min reperfusion), AKI 30 group (renal ischemia, 30 min reperfusion) and AKI 40 group (renal ischemia, 40 min reperfusion). Mice serum and 24-h urine were collected on the 8th, 9th, 10th, 14th, and 28th days for urinary protein, serum creatinine (Scr) and blood urea nitrogen (BUN) analysis. HE staining, transmission electron microscopy (TEM), Masson staining, Q-PCR, Western Blot and immunohistochemistry were applied.

**Results:**

Serum Scr and BUN levels across all AKI groups at the 9th day were significantly higher (*P* < 0.05) than controls, with higher reperfusion groups maintaining that increase up to 28 days (*P* < 0.05). Compared with Ctr group, the urinary protein of the AKI 40 group significantly rose on the 9th day (*P* < 0.05), normalizing immediately on the 10th day (*P* < 0.05). In contrast, the AKI 30 group rose significantly on the 14th day (*P* < 0.05) maintaining elevated levels for two weeks (*P* < 0.05). HE staining demonstrated ischemia-dependent renal tissue damage was aggravated in the mild to aggravated AKI groups. Mesangial proliferation, glomerulosclerosis, and tubulointerstitial pathology were also significantly increased in these groups (*P* < 0.05). Masson staining further showed that glomerular, renal tubular, and interstitial collagen were increased by ischemia in a time-dependent manner. Transmission EM additionally that podocytes of the mild to severe AKI groups displayed extensive fusion, exfoliation and GBM exposure. Synaptopodin, Nephrin, and CD2AP mRNA and protein expression demonstrated ischemic time-dependent decreases, while the TRPC6 was increased. There was a significant difference in the levels of Synaptopodin, Nephrin, CD2AP, and TRPC6 between the mild and severe AKI groups (*P* < 0.05).

**Conclusions:**

1) During the AKI process mice podocyte injury, proteinuria and the subsequent progression into chronic renal fibrosis is observed.2) Podocyte injury may be one of the causes of ischemia-reperfusion acute kidney injury and post-injury fibrosis.

**Electronic supplementary material:**

The online version of this article (10.1186/s12882-019-1298-x) contains supplementary material, which is available to authorized users.

## Background

Chronic kidney disease (CKD), a disease involving irreversible renal dysfunction or structural damage caused by heterogeneous disease pathways [1], has become a public health issue worldwide. The prevalence of CKD in Chinese adults has reached as high as 10.8% [2]. Delaying the progression of CKD has become one of the most important topics in the study of kidney disease. Acute kidney injury (AKI) is a critical illness involving pathological lesions as seen across a variety of clinical disciplines. The incidence rate in Chinese hospitalized patients is 5–7%, while the mortality rate in critically ill patients is 50%. About 50% of patients live with permanent renal dysfunction, which not only impacts patient prognosis, but also creates a heavy medical burden [3]. In the past, AKI was considered to be a self-healing disease. Recent studies, however, have emphasized that the recovery of renal function in patients who survive AKI is often incomplete. AKI can be sustained as CKD, and may even progress to end-stage renal disease (ESRD) [4]. In a meta-analysis in 2009, 48 studies were combined to identify 7017 AKI patients. This study concluded that AKI is a direct cause of CKD [5]. For a long period of time, clinical practice had followed the principle of symptomatic treatment. To date, no effective treatment measures exist to reduce tissue damage, promote repairing or prevent the occurrence of chronic fibrosis of the kidney. Thus, preventing the progression of AKI to CKD has become the focus of much of the international kidney disease research community.

In our clinical work, we have observed during the follow-up, that patients with partial AKI gradually developed progressive and persistently aggravated proteinuria, which led to a decrease in Glomerular filtration rate (GFR). The studies of proteinuria after AKI are sparse, and prospective studies by Horne KL et al. [6] showed that proteinuria and albuminuria are actually very common and persistent after AKI. They additionally found so much so that even the early phase AKI can have significant long-term effects on renal function and proteinuria of general hospitalized populations. As Greenberg et al. [7] systematically evaluated the occurrence of post-AKI CKD in 346 children, the average follow-up period being 6.5 years (2–16 years), the combined incidence of proteinuria, hypertension, and long-term mortality after AKI were 13.2%, 6.6 and 17.6%respectively. In addressing proteinuria after AKI, many investigators have focused on the role of renal tubular and interstitial injury during AKI. More recently, however, the involvement of podocyte injury as an etiological factor of progressive proteinuria and GFR decline (which can persist for several years) has become a subject of interest.

Due to persistent tissue ischemia and hypoxia, injury of vascular endothelial cells, reduction of microvascular beds, and inhibition of angiogenesis, AKI can often lead to the release of fibrogenic factors, fibrous tissue hyperplasia, and renal fibrosis. At present, the research on the pathophysiology and related mechanisms of AKI progression to CKD has mainly focused on microvascular endothelial cell injury, persistent inflammatory response, and abnormal activation of renal tubular epithelial cells. Despite the effort, however, none of the aforementioned mechanisms can explain the emergence of progressive and persistent aggravated proteinuria during renal chronicity after AKI. Pathophysiology studies of the impact of chronic post-AKI on podocytes, a critical glomerular filtration barrier, have rarely been carried out. Y Peng et al. [8] have shown that Toll-like receptor 2 (TLR2) is excessively activated in glomerular endothelial cells and podocytes in a mouse model of sepsis AKI. Double immunofluorescence analysis revealed that TLR2 and synaptopodinare co-localized in glomerular podocytes, meanwhile Bax and Caspase-3 expression are increased in the glomeruli, suggesting that TLR2 may mediate post-AKI podocyte apoptosis. Studies on lupus podocyte disease have found a high incidence of AKI in patients with lupus podocyte disease [9], suggesting that podocytes may participate in the development and progression of AKI. In this study, a model of chronic renal fibrosis following acute kidney injury (AKI) in BALB/c mice was established to investigate the role of podocyte injury in both AKI and the process of chronic renal ischemia following AKI. We propose that podocyte injury may be one of the causes of ischemia-reperfusion acute kidney injury and post injury fibrosis.

## Methods

### Materials

Fifty BALB/C mice (SPF level, 25 Male and 25female) aged 16 weeks (25–28 g) were provided by Shanghai Slac Co. (production license number: SCXK (Shanghai) 2012–0002). Raised in the SPF animal room of Fujian Medical University. Type of cage: rectangular squirrel cage (size as295 × 190 × 125 mm), cage material: polypropylene plastic box; light/dark: 12/12 h; temperature: 20–22 degrees celsius; mice free to drink water (edible water: filtered tap water); free to eat bedding material: high pressure corn kernel (SPF grade mouse material provided by Shanghai Slack Company). Antibodies included rabbit anti-mouse Nephrin, rabbit anti-mouse Synaptopodin polyclonal antibody, rabbit anti-mouse TRPC6 (Abcam), rabbit anti-mouse CD2AP (CST), β-actin (Santa cruz), secondary antibody (China JinshanJinqiao).

### Establishment of mouse ischemia-reperfusion model

Fifty BALB/C mice were randomly divided into control group (Ctr), sham group (sham), ischemia 20 min reperfusion group (AKI 20 group), ischemia 30 min reperfusion group (AKI 30 group) and 40 min reperfusion group (AKI 40 group), with 10 mice in each group. The mice were fasted 8 h before surgery and intraperitoneally anesthetized with3% sodium pentobarbital (1.0–1.5 ml/kg). A 1.5–2.0 cm incision was made along the ventral midline and the renal artery was isolated while the left renal artery was clamped with a micro-artery clip. It was observed that the kidneys turned pale from bright red. The arterial clamp was removed after 20, 30, or 40 min as assigned by group, followed by blood flow restoration. Model success and judgment: successful modeling during surgery: After the left kidney pedicle was clipped during surgery, the kidneys turned from bright red to pale; after the arterial clip was removed and blood perfusion was restored, the kidneys quickly turned from pale to the original color of bright red. And 1-3 h after surgery, the mice were found awakened, then gradually returning to normal activities. If modeling was unsuccessful: 5 min after resuming the blood flow of the kidneys, the kidneys did not return to the normal color; it was possible that the surrounding tissues or organs had been damaged during the operation; or the mice did not wake up or died after 1–3 h. The control group (Ctr) received no treatment and in the sham group, the left renal artery was dissociated but not blocked. On the 8th day following surgery, the right kidneys were removed from the mice in all groups [10]. The project design was conducted in line with scientific and ethical principles (Animal welfare meets the requirements of the Animal Ethics Committee of Fujian Medical University). And the study was approved by The Animal Ethics Committee of Fujian Medical University (approval number: 2017–062).

### Determination of mouse urinary protein and renal function

On the 8th, 9th, 10th, 14th, and 28th days following surgery, the mice were collected for 24 h urine collection in a metabolic cage. Urine was centrifuged at 1000 g for 10 min, and the supernatant was stored at − 80 °C. Blood was collected using a capillary glass tube,0.5 ml each, and 1000 g centrifugation for 10 min was performed to isolate the supernatant. Twenty-four urinary protein, serum creatinine (Scr), and urea nitrogen (BUN) were measured by automatic biochemical analyzer.

### HE staining and Masson staining

Mice were abdominally anesthetized with 3% sodium pentobarbital (1.0–1.5 ml/kg) and the left kidneys were excised, fixed in 10% formalin and embedded in paraffin. Three μm serial sections were cut, routine de-waxed, hydrated, and stained by hematoxylin-imidine Red (HE) or Masson stain. After the experiment, 3% pentobarbital sodium 2 ml/kg (60 mg/kg) were intraperitoneally injected, 10 min later, mice were sacrificed by cervical dislocation.

### Kidney pathological damage scoring

Routine HE staining and Masson staining were performed on the kidney tissue and renal pathology scores were obtained after observation under light microscope according to the following criteria:

- glomerular mesangial proliferation: no mesangial proliferation = 0 point; mild mesangial proliferation = 1 point; moderate mesangial proliferation = 2 points; severe mesangial proliferation = 3 points.
- Degree of glomerular sclerosis: No glomerular sclerosis = 0 point; Glomerular sclerosis rate < 25% = 1 point; 25–50%,=2 points;> 50%,=3 points.
- Renal tubulointerstitial score (RTIS) was determined according to four indicators: (1) degeneration and necrosis of renal tubule; (2) tubular atrophy; (3) infiltration of interstitial inflammatory cells; (4) interstitial fibrosis. According to the extent and severity of lesions, null, < 25, 25–50%, and > 50% were scored as 0, 1, 2, and 3, respectively.

### Transmission electron microscopy to assess glomerular and podocyte morphology changes

The renal cortex tissue was harvested, and 1 mm^3^ tissue blocks were cut in electron microscope fixed solution, dehydrated with alcohol-acetone, embedded in epoxy resin (618 embedding medium), sliced, and stained with uranyl acetate and lead citrate. The ultrastructure of podocytes and glomerular sclerosis were observed by transmission electron microscope.

### Immunohistochemistry

Paraffin sections were conventionally dewaxed and hydrated. The addition of 3% H2O2 was performed at room temperature for 10 min, followed by10 min × 3 PBS was, addition of the primary antibody at 50 μl/tablet, and incubation at 37 °C for 1 h. Sections were then washed with PBS for another 10 min × 3, followed by secondary antibody incubation at 37 °C for 30 min and final PBS rinse for 10 min × 3,. Diaminobenzadine was used as a chromogen for the immunoreactive signal and sections were incubated untilcolor developed. Sections were counterstained inhematoxylin followed by gradient dehydration, and were resin sealed. Sections devoid of primary antibody were used as negative controls. Under amicroscopic field of 200 magnification, five fields were randomly selected from each slice. The optical analysis (IOD) value was determined by image analysis system (Motic Images Advanced), and the protein expression level was expressed as the IOD value.

### Q-PCR

About 20 mg of kidney tissue were incubated in 1 ml Trizolfor homogenation. The lysate was then processed according to the instructions for total RNA extraction and reverse transcription (Transcriptor First Strand cDNA Synthesis Kit, Roche USA). The primer sequences and amplification length used for each gene are designed and synthesized by TaKaRa. Quantitative amplification was performed on an LightCycler® 96 Fluorescent PCR instrument using SYBR Green (FS Essential DNA Green Master, RocheUSA). The PCR reaction conditions were: pre-denaturation at 95 °C for 30s, denaturation at 95 °C for 5 s, annealing at 60 °C for 30s, and extension at 72 °C for 30s. GAPDH was used as an internal reference gene to analyze the relative mRNA level. The result was shown as a 2-ΔΔCt value.(PCR primers and product sizes are further summarized in Additional file 1: Table S1).

### Western-blot kidney tissue Synaptopodin, Nephrin, CD2AP and TRPC6 protein levels

RIPA lysate was used to extract total protein from kidney tissues and the protein content was determined according to Braford protein quantification kit instructions. Proteins were separated by 10% SDS-PAGE, transferred to membranes, and incubated in 5% skim milk powder at room temperature (25 °C) for 30 min. Primary antibodies were added at the following concentrations: Nephrin (1:400), CD2AP (1:1000), Synaptopodin (1:2000), ant TRPC6 (1:1000). Membranes were incubated in antibody solution at 37 °C for 1.5 h, rinsed in PBS for 10 min × 3, and incubated in secondary antibody (1: 2000) at 37 °C for 1 h. Membranes were rinsed a final time with PBS for 10 min × 3, exposed to light, and protein was visualized by White/Ultraviolet Transilluminator system. Beta-аctinprotein was used as internal reference and target protein/β-аctin (OD) ratio was used to indicate target protein levels.

### Statistical analysis

All data were expressed as mean ± standard deviation $\left(\overline{x}\cdot \pm S\right)$. Data analysis was performed using SPSS19.0 statistical analysis software. One-way ANOVA was used for comparison among multiple groups. Subsequently, between group differences were compared byLSDtest. *P* < 0.05 indicates statistical difference.

## Results

Fifty BALB/C mice were successfully modeled. On the 28th day, in AKI 20 group, Ctr group, sham operation group, AKI 30 and AKI 40 group, ten mice survived in each group. In the AKI 20 group, the Ctr group and sham groups, mice had bright hair and good activity, while in the AKI 30group and AKI 40 group, mice had dull hair and reduced activity.

### Biochemical and urine analysis

Compared with the Ctr group, serum Scr and BUN levels in the AKI 20 group, AKI 30 group, and AKI 40 group on the 9th day were significantly higher (*P* < 0.05). Compared with the 9th day, the Scr and BUN levels of the 10th day were decreased across all three groups (*P* < 0.05). In AKI 30 and AKI 40 group specifically, Scr and BUN increased further on the 14th and 28th days (*P* < 0.05), though the AKI 20 group, Ctr group and sham groups did not (*P* > 0.05). Compared with Ctr group, urinary protein was significantly increased on the 9th day after injury in the AKI 40 group (*P* < 0.05), with the spike immediately diminished the next day (*P* < 0.05) with no subsequent fluctuations. Compared with Ctr group, the urine protein level in AKI 30 group was also significantly increased, though on the 14th day (*P* < 0.05) and 28th days (*P* < 0.01). In contrast, no significant difference was observed among the AKI 20 group and all controls (*P* > 0.05). The results are further summarized in Table 1 and Additional file 2: Table S2.

**Table 1:** Changes in urinary protein, BUN and Scr in each group $\overline{x}\kern0.5em \cdot \pm \kern0.5em s$, *n* = 10)

	Group	D8	D9	D10	D14	D28
UPE (mg/d)	Ctr	1.47 ± 0.11	1.43 ± 0.18	1.45 ± 0.16	1.43 ± 0.14	1.46 ± 0.21
	Sham	1.42 ± 0.18	1.49 ± 0.16	1.40 ± 0.15	1.42 ± 0.15	1.48 ± 0.20
	AKI 20	1.43 ± 0.15	1.53 ± 0.24	1.48 ± 0.17	1.50 ± 0.12	1.52 ± 0.15
	AKI 30	1.46 ± 0.13	1.86 ± 0.38	1.97 ± 0.22	3.64 ± 1.12^*^	10.88 ± 2.44^**^
	AKI 40	1.38 ± 0.20	3.47 ± 0.63^*^	2.52 ± 0.26^*^	1.54 ± 0.33	1.55 ± 0.18
BUN (mmol/L)	Ctr	11.41 ± 1.22	12.28 ± 1.01	10.99 ± 0.43	12.37 ± 1.29	10.93 ± 1.20
	Sham	12.07 ± 1.31	10.92 ± 1.26	11.74 ± 0.55	12.49 ± 1.31	11.56 ± 1.47
	AKI 20	11.89 ± 1.36	27.30 ± 2.32^*^	15.13 ± 1.11^*^	12.20 ± 1.24	11.12 ± 1.13
	AKI 30	10.84 ± 1.47	28.48 ± 2.01^*^	17.36 ± 1.32^*^	25.45 ± 2.99^*#^	28.77 ± 1.23^*#^
	AKI 40	12.35 ± 1.41	30.19 ± 2.62^*#^	25.27 ± 2.53^*#^	28.66 ± 1.78^*#^	37.48 ± 2.15^*#Δ^
Scr (umol/L)	Ctr	7.41 ± 0.82	7.20 ± 0.81	6.91 ± 0.63	7.03 ± 0.72	6.89 ± 0.75
	Sham	7.03 ± 0.71	7.19 ± 0.98	7.17 ± 0.81	6.99 ± 0.76	7.11 ± 0.87
	AKI 20	7.12 ± 0.80	16.39 ± 1.84^*^	13.88 ± 1.21^*^	7.78 ± 1.26	7.35 ± 1.18
	AKI 30	7.81 ± 0.67	16.72 ± 1.56^*^	12.96 ± 1.43^*^	15.26 ± 1.29^*#^	22.34 ± 1.17^*#^
	AKI 40	7.39 ± 0.88	16.58 ± 2.07^*#^	23.22 ± 2.38^*#^	28.97 ± 2.92^*#Δ^	28.04 ± 1.09^*#Δ^

UPE, urinary protein excretion; BUN, blood urea nitrogen; Scr, serum creatinine. ^*^P < 0.05 vs Ctr, ^**^P < 0.01 vs Ctr, ^#^*P* < 0.05 vs AKI 20*,* ^Δ^*P* < 0.05 vs AKI 30

### Kidney pathological lesion scoring

**Fig. 1:**
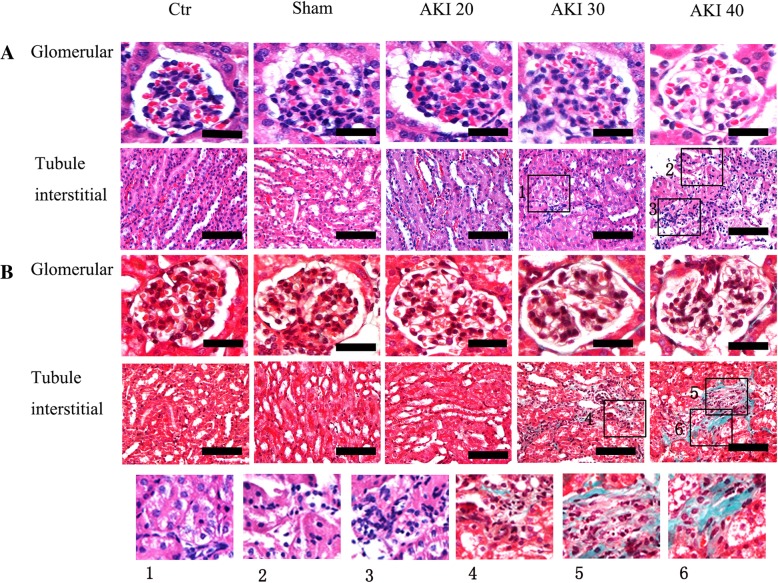
**a**: HE **b**: Masson Histology of kidneys on the 28th day after renal injury. AKI 20 group (mice underwent renal artery clamping for 20 min followed by contralateral nephrectomy 8 days after injury), AKI 30 group (mice underwent renal artery clamping for 30 min followed by contralateral nephrectomy 8 days after injury), AKI 40 groups (mice underwent renal artery clamping for 40 min followed by contralateral nephrectomy 8 days after injury). Kidneys were collected, and 3-mm paraffin sections were stained with hematoxylin and eosin (HE) stain and Masson’s trichrome. Scale bar =50 μm. Higher magnification: 1. Tumor tubular epithelial cells are swollen and the particles denote vacuolar degeneration. 2. Partial tubular epithelial cell damage with brush border detachment. Part of the renal tubular epithelial cells are necrotic. 3. Infiltration of renal interstitial lymphoid mononuclear cells. Renal tubular epithelial cells are swollen and the particles denote vacuolar degeneration. 4. Renal interstitial exchange for a small amount of lymphocyte mononuclear cell infiltration. 5. Renal interstitial infiltration of multiple lymphoid mononuclear cells with interstitial fibrosis. 6. Renal interstitial fibrosis, tubular atrophy

HE staining showed that the glomerular volume in the Ctr, Sham and AKI 20 group was nominally increased, the capillary fistula was well-opened, the focal mesangial cells were slightly hyperplastic, and the renal tubules and interstitium were basically normal. In the AKI 30 group, glomerularhyperplasia, hypertrophy, mesangial cell hyperplasia, focal segmental glomerular sclerosis, tubular vacuolar degeneration, mild interstitial inflammatory cell infiltration, and focal tubular atrophy were observed. Additionally, the glomerular basement membrane demonstrated matrix deposition and glomerular ischemic contraction or sclerosis were also apparent. In the AKI 40 group, extensive tubular denaturation and vacuolar degeneration of renal tubular epithelial cells was seen. Multifocal glomerular epithelial cells, brush border detachment with segmental basement membrane was exposed and no tubular epithelial cell regeneration was seen. A large number of red blood cell casts were visible in the lumen, and multifocal lymphoid and mononuclear cell infiltration in the renal interstitium, as well as tubular basement membrane and matrix-filled perivascular vessels were observed. Masson staining additionally showed no blue staining in the glomeruli, renal tubules, or renal interstitium in either of the Ctr, Sham, and AKI 20 groups. On the other hand, a small amount of platelet-like tissue staining was observed around the glomerulus and interstitium in the AKI 30 group. The level of collagen in glomeruli, renal tubules and interstitial collagen in the AKI 40 group was significantly higher than the AKI 30 group(*P* < 0.05), which affected even flake-like tissue (Fig. 1).

Kidney pathological scores showed no significant difference between glomerular mesangial proliferation and glomerulosclerosis scores and renal tubulointerstitial scores of the Ctr, Sham, and AKI 20 groups (*P* > 0.05). Conversely, all three scores in the AKI 30 and AKI 40 groups were significantly higher than controls (*P* < 0.01). Among the AKI 30 and AKI 40 groups, the renal tubulointerstitial scores were significantly different (*P* < 0.05)(Table 2).

**Table 2:** Comparing renal pathological damage in each group (pathological score, $\overline{x}\cdot \pm s$, *n* = 10)

Group	Ctr	Sham	AKI 20	AKI 30	AKI 40
Mesangial hyperplasia score	0 ± 0	0 ± 0	0.67 ± 0.51^**^	1.67 ± 0.82^**^	1.33 ± 0.52^**^
Glomerular sclerosis score	0 ± 0	0.17 ± 0.41	0.50 ± 0.55	1.17 ± 0.75^**^	1.33 ± 0.52^**^
Tubule interstitial score	0.5 ± 0. 55	0.83 ± 0.75	1.00 ± 0.63	6.17 ± 1.17^**^	9.50 ± 1.05^**# #^

^**^P < 0.01 vs Ctr, ^# #^*P* < 0.01 vs AKI 30

### Transmission electron microscopy (TEM) to evaluate changes in glomerular and podocyte morphology

**Fig. 2:**
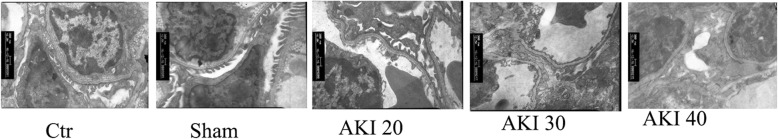
Glomerular ultrastructure of kidneys on the 28th day after renal arterial injury. Control, Sham and AKI 20: No foot process effacement or flattening was seen. AKI 30: extensive foot process fusion was detected. AKI 40: extensive podocyte foot process stripping and GBM exposure were detected

Transmission electron microscopy showed that the podocytes in the Ctr, Sham, and AKI 20 groups were intact, with clear structures and no observable foot process fusion. In the AKI 30 group, however, the podocytes were found extensively fused in the foot processes and completely detached in the AKI 40 group, exposing GBM (Fig. 2).

### Comparison of changes in the expression of podocyte functional proteins

**Fig. 3:**
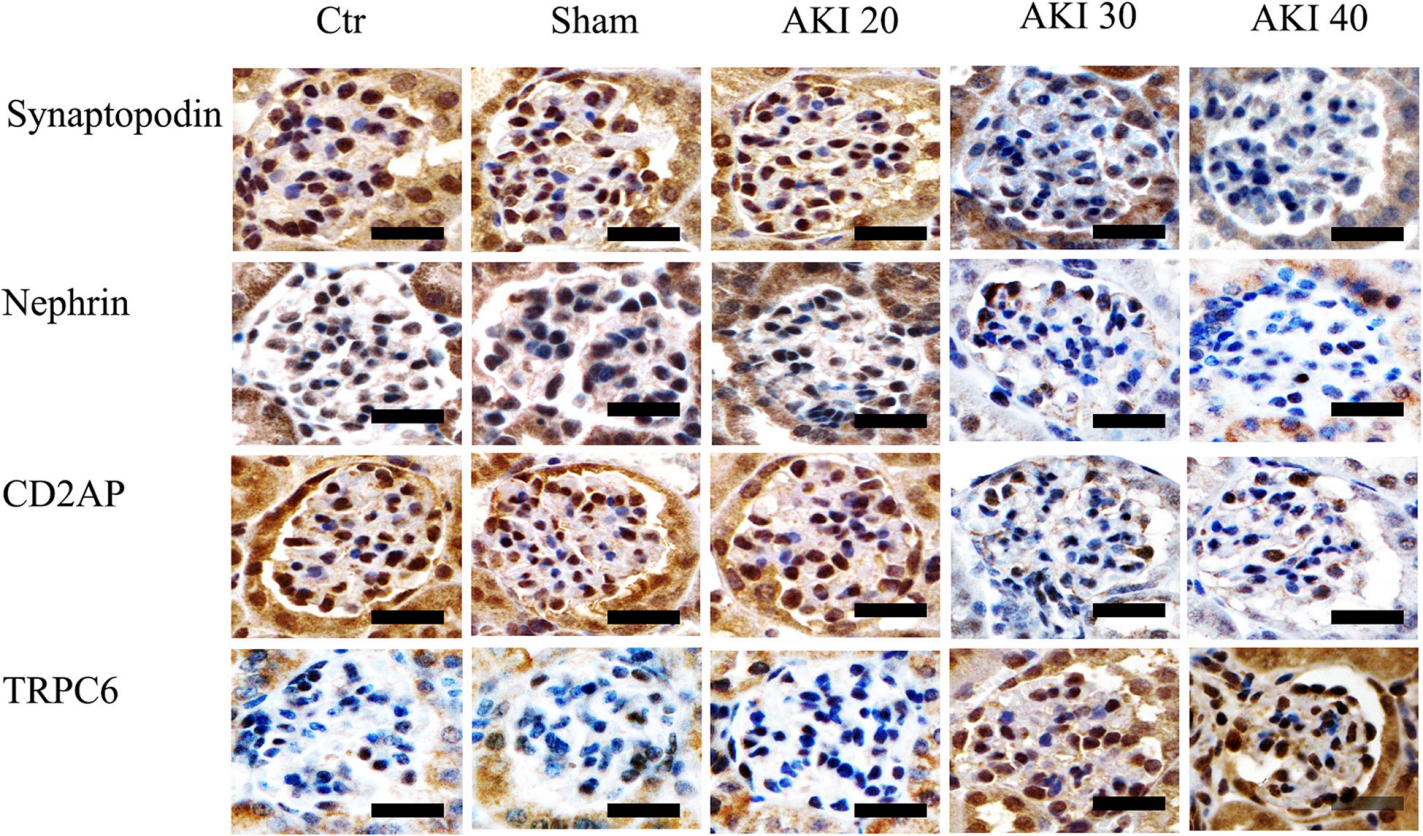
Expression of Synaptopodin, Nephrin, CD2AP, and TRPC6 protein by immunohistochemical staining. AKI 20 group (mice underwent renal artery clamping for 20 min followed by contralateral nephrectomy 8 days after injury), AKI 30 group (mice underwent renal artery clamping for 30 min followed by contralateral nephrectomy 8 days after injury), AKI 40 groups (mice underwent renal artery clamping for 40 min followed by contralateral nephrectomy 8 days after injury). Scale bar =50 μm

Immunohistochemical results showed substantial Nephrin and Synaptopodin immunoreactivity in the cytoplasm of the Ctr, Sham, and AKI 20 groups, with only small amount of immunoreactivity in the podocyte cytoplasm of the AKI 30 and AKI 40 groups (Fig. 3).

The immunohistochemistry results of CD2AP demonstrated significant immunoreactivity in the podocyte cytoplasm and perinucleus in the Ctr, Sham, and AKI 20 group, while only a small amount of perinuclear signal was detected in the AKI 30 group. Further, only a small amount of almost no signal was detected either in the perinuclear region or cytoplasm of the podocyte in the AKI 40 group (Fig. 3).

**Fig. 4:**
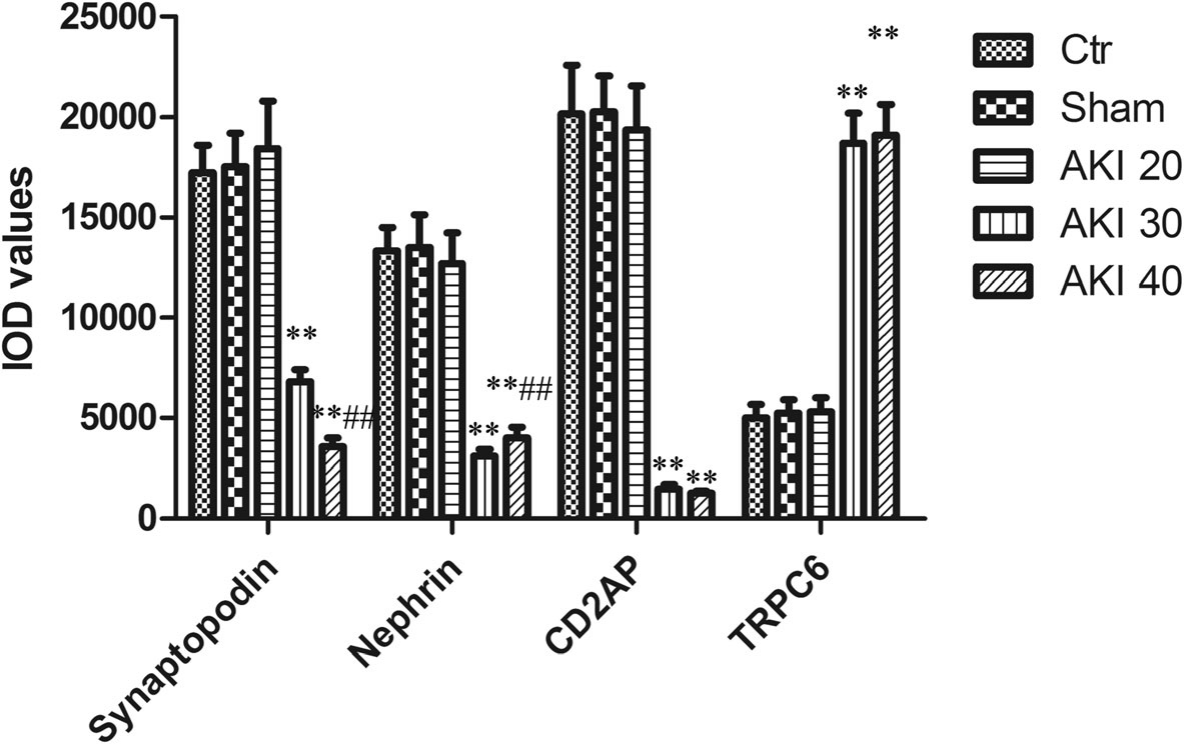
Comparison of Synaptopodin, Nephrin, CD2AP, and TRPC6 protein by immunohistochemical staining in each group (IOD values). Data are $\overline{x}\cdot \pm S$ (*n* = 10). **P < 0.01 versus Ctr, ^##^*P* < 0.01 versus AKI 30

TRPC6 immunohistochemistry showed almost no positive signal in the podocyte cytoplasm in the Ctr, Sham, and AKI 20 groups, though the AKI 30 and AKI 40 groups demonstrated a robust positive signal in the podocytes cytoplasm (Fig. 3). Immunohistochemistry analysis showed that compared with that of Ctr group, the expression of Synaptopodin, Nephrin and CD2AP in the AKI30 group was significantly decreased (*P* < 0.01), while the expression of TRPC6 was significantly increased (*P* < 0.01). Compared with the AKI 30 group, the expression of Synaptopodin, Nephrin and CD2AP in the AKI 40 group was significantly decreased (*P* < 0.05), while the expression of TRPC6 was significantly increased (*P* < 0.05). In neither of the above indicators was a significant difference detected among the Ctr, Sham or AKI 20 groups(*P* > 0.05), (Fig. 4).

**Fig. 5:**
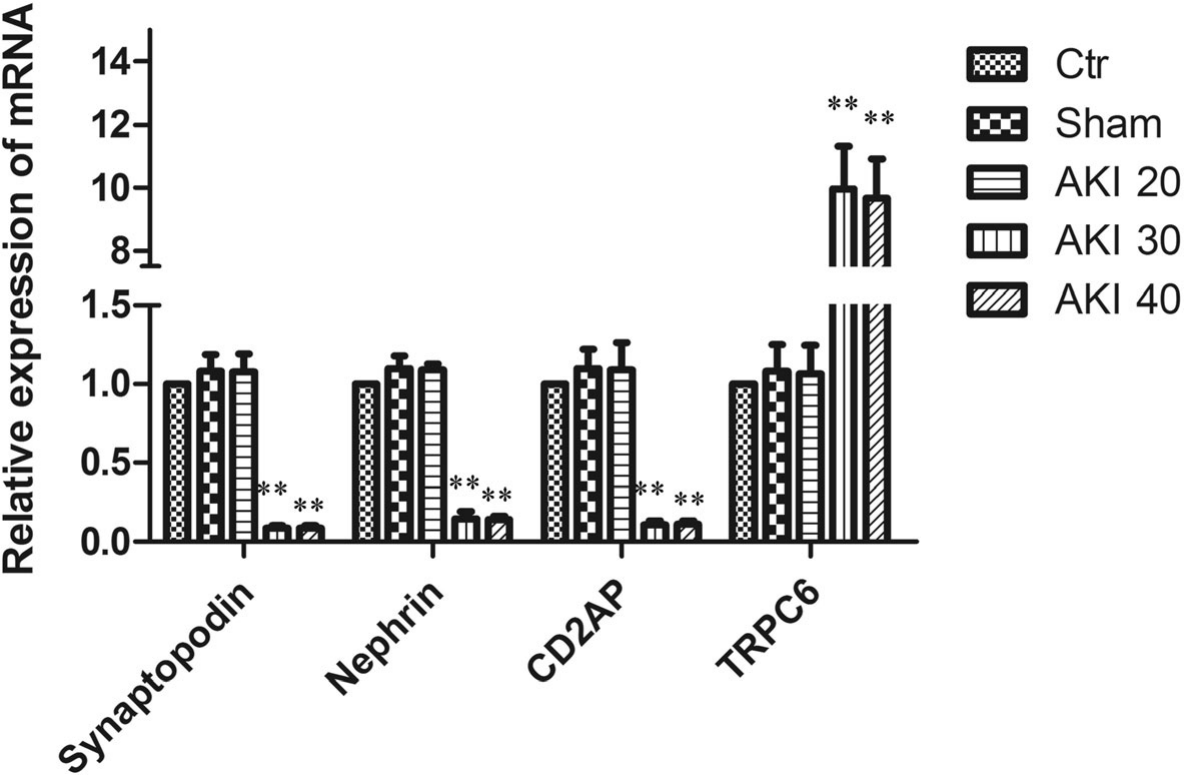
Comparisonof Synaptopodin, Nephrin, CD2AP and TRPC6 mRNA in each group. Relative expression of mRNA by Q-PCR. Data are $\overline{x}\cdot \pm S$ (*n* = 10). **P < 0.01 versus Ctr

**Fig. 6:**
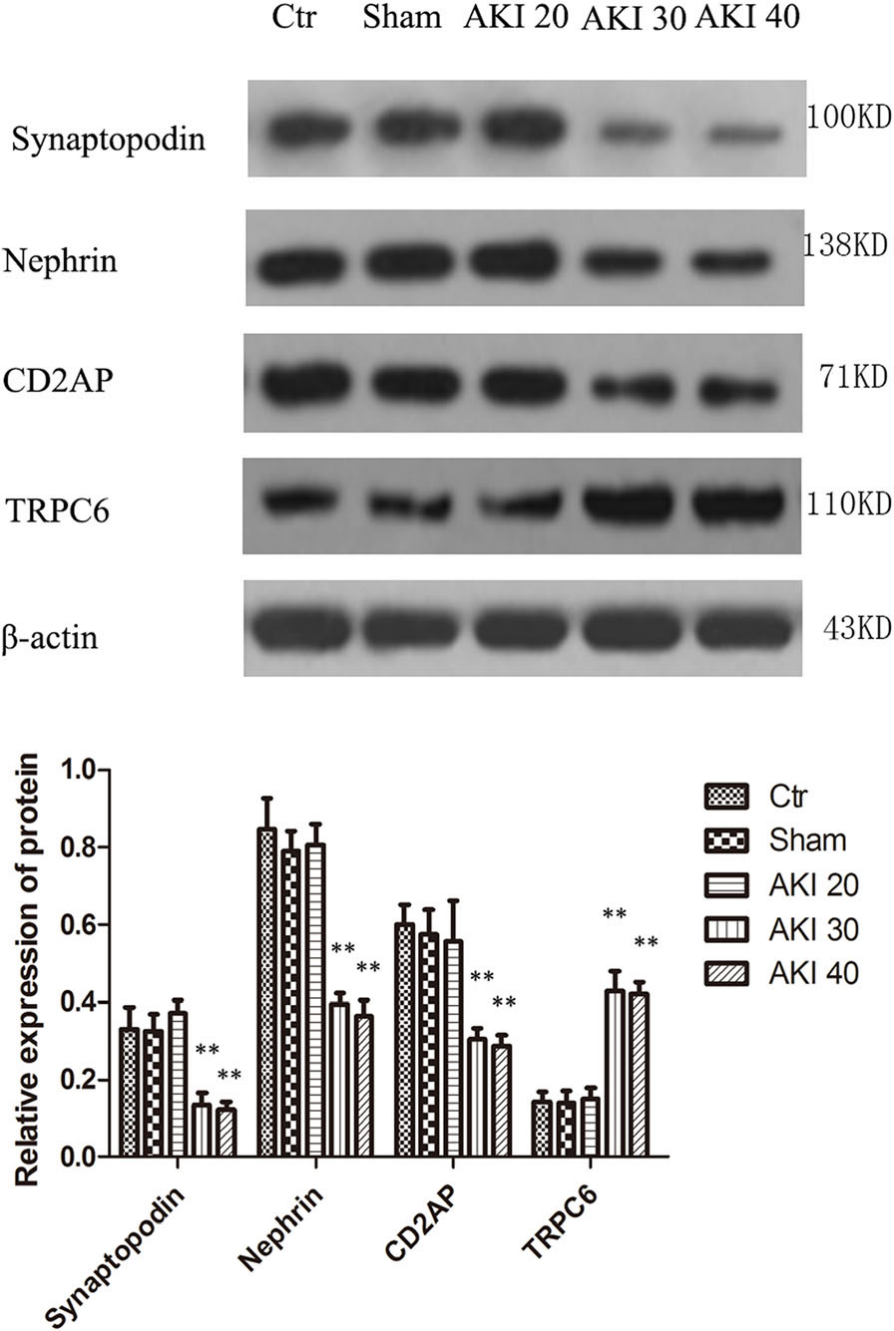
Levels of Synaptopodin, Nephrin, CD2AP, and TRPC6 protein in kidneys of each group measured by Western-blot. Upper: Electrophoretic film exposure images, Lower: Optical density analysis of electrophoretic film exposure images, showing the target protein level as a ration of β-аctin. Data are $\overline{x}\cdot \pm S$ (*n* = 10). **P < 0.01 versus Ctr

Q-PCR and Western Blot further showed that the mRNA and protein expressions of Synaptopodin, Nephrin, and CD2AP decreased by ischemia in a time-dependent manner, while the expression of TRPC6 mRNA and protein increased in a time-dependent manner. Compared with controls, mRNA and protein of Synaptopodin, Nephrin, and CD2AP in the AKI 30 group were significantly decreased (*P* < 0.01), whileTRPC6 mRNA and protein were significantly increased (*P* < 0.01). Compared with AKI 30 group, mRNA and protein expression of Synaptopodin, Nephrin, and CD2AP were significantly decreased in the AKI 40 group (*P* < 0.05), whileTRPC6 mRNA and protein were significantly increased (*P* < 0.05). There was no statistical difference in the expression of either marker among either the AKI 20 group, Sham group or Ctr group (*P* > 0.05), (Figs. 5-6).

## Discussion

The global prevalence of CKD in the world continues to grow. The World Health Organization (WHO) estimates that CKD caused 864,225 deaths worldwide in 2012alone, accounting for 1.5% of the global death toll, and this figure is rising continually [11]. AKI not only boasts a high mortality rate, but also a high risk of developing into CKD. Therefore, AKI has become a global public health concern in recent years [12, 13]. According to the prospective studies of Mammen et al. [14] on children with AKI, the incidence of CKD during a 3-year follow-up period was 4.5, 10.6, and 17.1% for pediatric patients with AKI stage 1, 2, and 3, respectively. Additionally, they report that up to 46.8% of pediatric patients suffered from hypertension, microalbuminuria, or a mild decline in GFR. It has been suggested that these children are at higher risk of developing CKD due to the incidence of CKD and complications that induce CKD (hypertension, diabetes, cardiovascular disease, etc.). However, CKD rates among children are not high, suggesting that a more precursory condition such as AKI may instead be associated with the occurrence of proteinuria and subsequent CKD.

In this study, podocyte injury, extensive foot process fusion, and decreased expression of the functional proteins nephrin, CD2AP, and synaptopodin were observed in BALB/c mice after renal ischemia at 30 min after reperfusion. Increased expression of TRPC6, progressive aggravation of proteinuria, decreased renal function, and ultimately glomerular fibrosis were also observed. We propose that podocyte injury may be one of the underlying causes of ischemia-reperfusion acute kidney injury and post injury fibrosis.

Podocyte injury is a major cause of proteinuria and the progression of numerous glomerular diseases [15, 16]. The podocyte is a highly specific, terminally differentiated, epithelial cell located on the outermost layer of the glomerular basement membrane (GBM). Together with GBM and glomerular endothelial cells, it forms a glomerular filtration barrier. According to the severity and duration of injury factors, podocytes may undergo foot fusion, podocyte hypertrophy, epithelial mesenchymal transition (EMT),apoptosis or successive shedding. When the injury factor is strong and long-lasting, podocytes may skip the progressive injury response and directly enter into a severely injured state when the relevant signal pathway is activated. At present, the research on the pathophysiology of the progression of AKI to CKD is still lacking. The establishment of a relevant animal model is a crucial step in understanding the mechanisms of AKI to CKD progression. A large number of animal models have been developed to simulate the clinical progression of AKI, however each animal model is usually created using a specific method that limits the interpretation and generalizability of the specific model [17]. Ischemia-reperfusion induced acute kidney injury is widely used as a model for mice with AKI, but the results are usually quite variable with high, often unreported mortality. IRI models have different styles, each with their own natural processes of renal dysfunction and histopathology. Not all ischemia-reperfusion induced variants are suitable for studying the progression from AKI to CKD and post-fibrosis. Mingjun Shi et al. [18] used an animal model to longitudinally examine the progression of CKD after AKI. Mice underwent bilateral ischemia-reperfusion injury or unilateral nephrectomy plus contralateral ischemia-reperfusion injury and were followed up for 20 weeks after the single AKI episode. They believe that more severe AKI is associated with a more serious long-term prognosis, including higher mortality and incidence of CKD. Nathalie Le Clef et al. [19] demonstrate in C57Bl/6J mice, by both histology and gene expression, that unilateral ischemia-reperfusion without contralateral nephrectomy is a very robust model to study the progression from acute renal injury to longtermtubulo-interstitial fibrosis. The model of severe bilateral renal ischemia-reperfusion and that of unilateral renal ischemia-reperfusion with concurrent contralateral nephrectomy have higher mortality, while as for the model of unilateral renal ischemia-reperfusion without contralateral nephrectomy, the degree of sclerosing of the glomerulus was mild, the pathological manifestations of chronic renal fibrosis not occurring until after 6–12 weeks. To induce more severe AKI, BALB/c mice undergo renal pedicle clamping for 30 min followed by contralateral nephrectomy 8 days after injury. This allows functional assessment of renal recovery after injury with 90–100% survival. After 4 weeks, the kidneys showed stable chronic fibrosis. Early post-injury tubular damage as well as post injury fibrosis are highly consistent using this model [10]. So we improved the chronic kidney injury models in mice. Our study found that kidney health after AKI in mice is closely related to the duration of ischemia-reperfusion injury. Specifically, mice with reperfusion injury after 20 min of ischemia demonstrated a good prognosis, where the acute injury of the kidney has been almost completely repaired. Ischemia followed by 30 min reperfusion-injured mice gradually developed deteriorating proteinuria and decreasing renal function. Pathological indicators showed growth in glomerular mesangial proliferation, glomerulosclerosis score and interstitial tubulointerstitial score. Glomeruli, renal tubules, and interstitial kidney collagen levels were also notably increased. Transmission electron microscopy further revealed extensive podocyte foot fusion and the expression of Nephrin, CD2AP, and Synaptopodin in podocytes fell. The expression of TRPC6 increased, on the other hand. Finally, chronic fibrosis was observed in the kidneys in the mild to severe injury groups. In the severe injury group, significant tubular and renal interstitial fibrosis, and glomerular ischemia were rapidly apparent and renal function rapidly declined. Therefore, the model of left kidney ischemia (30 min reperfusion injury) and right kidney resection can clinically simulate the progressive aggravation of proteinuria and GFR declinein patients after AKI. The model can be applied to study the molecular mechanisms of podocyte injury, proteinuria, and glomerular fibrosis after AKI in greater detail in future studies.

Nephrin was the first transmembrane globulin found to be specifically expressed on the podocyte septal membrane and plays an important role in maintaining the morphology of the septum [20]. Consequently, it serves as an early mark of podocyte injury [21]. In Nephrin knockout mice, the podocyte foot processes disappear, leading to septum deformity and podocyte apoptosis. Mesangial cell hyperplasia and sclerosis, glomerular basement membrane thickening, subendothelial band broadening, and severe proteinuria are also associated with Nephrin loss [22, 23]. CD2AP is distributed in the septal membrane of the podocyte and serves as a specialized intercellular connecting protein for the footpod of adjacent podocytes and as a cross-link for transmembrane adhesion proteins, such as podocin, Nephrin and NEPH1-2to maintain the slit diaphragm (SD) integrity of the podocyte and the formation and maintenance of the glomerular filtration barrier [24]. CD2AP damage can affect the stability of the cytoskeleton and interfere with the signal transduction pathway, interrupting cells and leading to the disappearance of the foot process fusion, destruction of SD integrity, and production of large amount of proteinuria [25]. The lack of CD2-associated protein (CD2AP) in mice increases podocyte apoptosis and results in glomerular sclerosis and renal failure. Tsuji K et al. [26] used powerful Helminion scanning microscopy (HIM) to examine the three-dimensional ultrastructure of CD2AP gene-deficient mice, finding various ultrastructural abnormalities of the glomeruli, including the appearance of numerous “bubble-shaped microprojections and the disappearance of the connective structure of podocytes. Synaptopodin is expressed in podocytes and telencephalon synapses with glomerular differentiation and maturation, and is one of the classic signs of podocyte maturation [27]. Expression of synaptopodin declines in a variety of kidney diseases, with changes in the structure and function of podocytes and in the production of proteinuria [28, 29]. Yu H [30] and other investigators demonstrated that Synaptopodin can limit the expression of TRPC6 on the podocyte surface to reduce proteinuria. Reduction of synaptopodin in a disease state can in turn alter the localization and activity of intracellular TRPC6 channels, exacerbating podocyte dysfunction. TRPC6 is a podocyte pore membrane protein that has only recently been discovered. Wiggins et al. [31] used plasmid transfection to increase the expression of podocyte TRPC6, confirming that TRPC6-mediated calcium influx is involved in the remodeling of the podocyte cytoskeleton, resulting in the disordered arrangement of cytoskeletal protein F-actin and lowered expression of Nephrin and Synaptopodin. Huang H et al. [32] further showed that TRPC6 signaling plays an important role in podocyte injury induced by TGF-β1. The results of this study indicate that the expression of Nephrin, CD2AP,Synaptopodin mRNA and protein decreased, the expression of TRPC6 mRNA and protein is elevated in mice with 30 min ischemia-reperfusion injury, along with the production of proteinuria and renal function decline, suggesting that cytoskeletal disorganization of the podocyte by Nephrin, CD2AP,Synaptopodinand TRPC6 may to some extent drive AKI and the progression of post- AKI chronic renal fibrosis.

Limitations of this study lies in that we did not perform protective intervention on podocytes during renal ischemia-reperfusion acute kidney injury in mice. We will conduct in vitro cell experiments to further study the mechanism of podocyte injury in the course of ischemia-reperfusion acute kidney injury.

In summary, our study shows that a BALB/C model of left kidney 30 min of reperfusion injury and right kidney resection can simulate the clinically condition of patients with progressive and persistent proteinuria, and decline in GFR after AKI. Our study shows that mice podocyte injury has been observed in AKI, that extensive podocyte fusion occurs in podocytes, and that expression levels of podocyte functional proteins including nephrin, CD2AP, and synaptopodin decrease while expression of TRPC6 increases, gradually aggravating proteinuria and subsequently hindering renal function, The pathology showed an increase in glomerular mesangial proliferation, glomerulosclerosis score, tubulointerstitial score, and the rising levels of glomerular, renal tubules, and interstitial collagen, eventually presenting chronic renal fibrosis. We deduce that podocyte injury may be one of the causes of AKI and chronic kidney disease after AKI.

## Conclusions

During the AKI process mice podocyte injury, proteinuria and the subsequent progression into chronic renal fibrosis is observed. Podocyte injury may be one of the causes of ischemia-reperfusion acute kidney injury and post-injury fibrosis.

## Additional files

Additional file 1: PCR primers and product sizes; PCR primers and product sizes of Nephrin, CD2AP, synaptopodin, TRPC6 and GADPH. (DOCX 16 kb)

Additional file 2: Changes in urinary protein, BUN and Scr in each group from D0 to D7 (^−^x ± s, *n* = 10) There is no significant difference of urine protein, Scr and BUN in AKI mouse model from day 0 to day 7. (DOCX 20 kb)

## Abbreviations

AKI 20 group: renal ischemia for 20 min then reperfusion
AKI 30 group: renal ischemia for30 minutes then reperfusion
AKI 40 group: renal ischemia for 40 min then reperfusion
AKI: Acute kidney injury
BUN: Blood urea nitrogen
CD2AP: CD2-associated protein
CKD: Chronic kidney disease
Ctr: Control group
EMT: Epithelial mesenchymal transition
ESRD: End-stage renal disease
GBM: Glomerular basement membrane
GFR: Glomerular filtration rate
HE: Hematoxylin-imidine Red
HIM: Helminion scanning microscopy
RTIS: Renal tubulointerstitial score
Scr: Serum creatinine
SD: Slit diaphragm
Sham: Sham group
TEM: Transmission electron microscopy
TLR2: Toll-like receptor 2
WHO: World Health Organization
