# Supplementary material for: The role of podocyte damage in the etiology of ischemia-reperfusion acute kidney injury and post-injury fibrosis
Source: BMC Nephrol. 2019 Mar 28;20:106. doi: 10.1186/s12882-019-1298-x (PMC6438002; doi:10.1186/s12882-019-1298-x)
Supplement: Supplementary file 1 — PCR primers and product sizes; PCR primers and product sizes of Nephrin, CD2AP, synaptopodin, TRPC6 and GADPH. (DOCX 16 kb) [file 12882_2019_1298_MOESM1_ESM.docx]

**Additional file 1: Table S1. PCR primers and product sizes**

| Target mRNA | Primer Sequence | PCR  product (bp) |
| --- | --- | --- |
| nephrin | F 5’-CAGGGAAGACAGCAACAAACAA-3’ | 197 |
|  | R 5’-CAGGTTTTCAGATAGAGCCCAGA-3’ |  |
| CD2AP | F 5’-AGGAATTCAGCCACATCCACA-3’ | 113 |
|  | R 5’-CGATCAATTCCAGTTCGTCCTC-3’ |  |
| synaptopodin | F 5’-GCTCGAATTCCGATGCAAATAAAC-3’ | 132 |
|  | R 5’-CAGGCCACAGTGAGATGTGAAGA-3’ |  |
| TRPC6 | F 5’-GCATCCAAAGCTCAGAGCATCA-3’ | 155 |
|  | R 5’-GCGATTGCATAAAGACCTTCAGAGA-3’ |  |
| GADPH | F 5’-TGTGTCCGTCGTGGATCTGA -3’ | 150 |
|  | R 5’-TTGCTGTTGAAGTCGCAGGAG-3’ |  |

PCR, polymerase chain reaction; CD2AP, CD2-associated protein; TRPC6, transient receptor potential channel 6; GAPDH, glyceraldehyde-3-phosphate dehydrogenase
