# Supplementary material for: The role of podocyte damage in the etiology of ischemia-reperfusion acute kidney injury and post-injury fibrosis
Source: BMC Nephrol. 2019 Mar 28;20:106. doi: 10.1186/s12882-019-1298-x (PMC6438002; doi:10.1186/s12882-019-1298-x)
Supplement: Supplementary file 2 — Changes in urinary protein, BUN and Scr in each group from D0 to D7 (−x ± s, n = 10) There is no significant difference of urine protein, Scr and BUN in AKI mouse model from day 0 to day 7. (DOCX 20 kb) [file 12882_2019_1298_MOESM2_ESM.docx]

**Additional file 2: Table S2. Changes in urinary protein, BUN and Scr in each group from D0 to D7 (‾x±s, n=10)**

|  | **Group** | **D0** | **D1** | **D2** | **D3** | **D7** |
| --- | --- | --- | --- | --- | --- | --- |
| UPE (mg/d) | Ctr | 1.45±0.19 | 1.46±0.15 | 1.45±0.17 | 1.44±0.11 | 1.43±0.16 |
|  | Sham | 1.46±0.20 | 1.47±0.22 | 1.45±0.18 | 1.43±0.21 | 1.46±0.15 |
|  | AKI 20 | 1.48±0.25 | 1.50±0.22 | 1.47±0.20 | 1.51±0.18 | 1.49±0.25 |
|  | AKI 30 | 1.50±0.23 | 1.52±0.26 | 1.48±0.24 | 1.53±0.30 | 1.51±0.24 |
|  | AKI 40 | 1.48±0.26 | 1.53±0.22 | 1.52±0.29 | 1.53±0.35 | 1.43±0.22 |
| BUN  (mmol/L) | Ctr | 11.55±1.31 | 12.01±1.24 | 11.34±1.30 | 12.13±1.52 | 11.07±1.25 |
|  | Sham | 12.18±1.44 | 12.45±1.55 | 11.96±1.38 | 12.06±1.40 | 11.76±1.36 |
|  | AKI 20 | 11.54±1.45 | 12.53±1.29 | 12.08±1.41 | 12.20±1.24 | 12.26±1.57 |
|  | AKI 30 | 11.08±1.36 | 12.15±1.46 | 11.15±1.44 | 12.24±1.53 | 11.17±1.35 |
|  | AKI 40 | 12.13±1.54 | 11.37±1.39 | 12.05±1.29 | 11.96±1.56 | 11.03±1.42 |
| Scr  (umol/L) | Ctr | 7.35±0.96 | 7.44±0.92 | 7.11±0.87 | 7.25±1.02 | 7.38±0.87 |
|  | Sham | 7.24±0.85 | 7.20±1.08 | 7.36±0.86 | 7.20±0.93 | 7.13±0.99 |
|  | AKI 20 | 7.06±0.91 | 7.15±0.77 | 7.11±0.92 | 7.33±1.05 | 7.28±1.02 |
|  | AKI 30 | 7.70±0.83 | 7.55±1.06 | 7.68±0.99 | 7.72±1.01 | 7.75±1.06 |
|  | AKI 40 | 7.35±0.76 | 7.49±0.81 | 7.65±0.90 | 7.42±0.78 | 7.27±0.83 |
| UPE, urinary proteinexcretion; BUN, blood urea nitrogen; Scr, serum creatinine. P>0.05 | | | | | | |
